# The role of the astrocyte in subarachnoid hemorrhage and its therapeutic implications

**DOI:** 10.3389/fimmu.2022.1008795

**Published:** 2022-09-29

**Authors:** Rong Li, Min Zhao, Di Yao, Xiangyue Zhou, Cameron Lenahan, Ling Wang, Yibo Ou, Yue He

**Affiliations:** ^1^ Department of Pediatrics, Tongji Hospital, Tongji Medical College, Huazhong University of Science and Technology, Wuhan, China; ^2^ Department of Neurosurgery, Tongji Hospital, Tongji Medical College, Huazhong University of Science and Technology, Wuhan, China; ^3^ Department of Neurology, Tongji Hospital, Tongji Medical College, Huazhong University of Science and Technology, Wuhan, China; ^4^ Department of Biomedical Sciences, Burrell College of Osteopathic Medicine, Las Cruces, NM, United States; ^5^ Department of Operating room, Tongji Hospital, Tongji Medical College, Huazhong University of Science and Technology, Wuhan, China

**Keywords:** subarachnoid hemorrhage, astrocyte, neurovascular unit, astrocyte activation, blood-brain barrier, glymphatic-meningeal lymphatic system, oxidative stress, cell death

## Abstract

Subarachnoid hemorrhage (SAH) is an important public health concern with high morbidity and mortality worldwide. SAH induces cell death, blood−brain barrier (BBB) damage, brain edema and oxidative stress. As the most abundant cell type in the central nervous system, astrocytes play an essential role in brain damage and recovery following SAH. This review describes astrocyte activation and polarization after SAH. Astrocytes mediate BBB disruption, glymphatic–lymphatic system dysfunction, oxidative stress, and cell death after SAH. Furthermore, astrocytes engage in abundant crosstalk with other brain cells, such as endothelial cells, neurons, pericytes, microglia and monocytes, after SAH. In addition, astrocytes also exert protective functions in SAH. Finally, we summarize evidence regarding therapeutic approaches aimed at modulating astrocyte function following SAH, which could provide some new leads for future translational therapy to alleviate damage after SAH.

## Introduction

Subarachnoid hemorrhage (SAH) is a clinical condition caused by rupture of intracranial blood vessels and direct inflow of blood into the subarachnoid space; the mortality rate of this condition is estimated to be approximately 50% (1). The pathophysiologic mechanism of SAH is complex and multifactorial. It was initially understood that astrocytes primarily play a supportive structural role as a type of glial cell in the CNS. However, a growing number of studies have found that astrocytes have multiple functions, such as blood−brain barrier (BBB) formation, neuroprotection, neuronal metabolism, defense against oxidative stress (OS), and facilitation of cell−cell crosstalk (2, 3). Increasing evidence has shown that astrocytes play a key role in SAH. Following SAH, astrocytes are activated and take on the A1 neurotoxic phenotype, in which they express C3 and Serping1 (4, 5). As an important component of the blood−brain barrier (BBB), astrocytes modulate BBB disruption through neuroinflammation after SAH. In addition, astrocytes have multiple interactions with other cells in SAH and might act in conjunction with them to influence SAH injury (6–9). Furthermore, astrocytes exert some neuroprotective functions in recovery stage after SAH. Some substances aimed at improving astrocyte functions have been studied; these treatments could potentially mitigate injury after SAH.

In this review, we summarize past research on the role of astrocytes in SAH and discuss translational implications for prospective treatments. 

## Astrocytes are involved in the pathophysiological process that occurs after SAH

**Figure 1:**
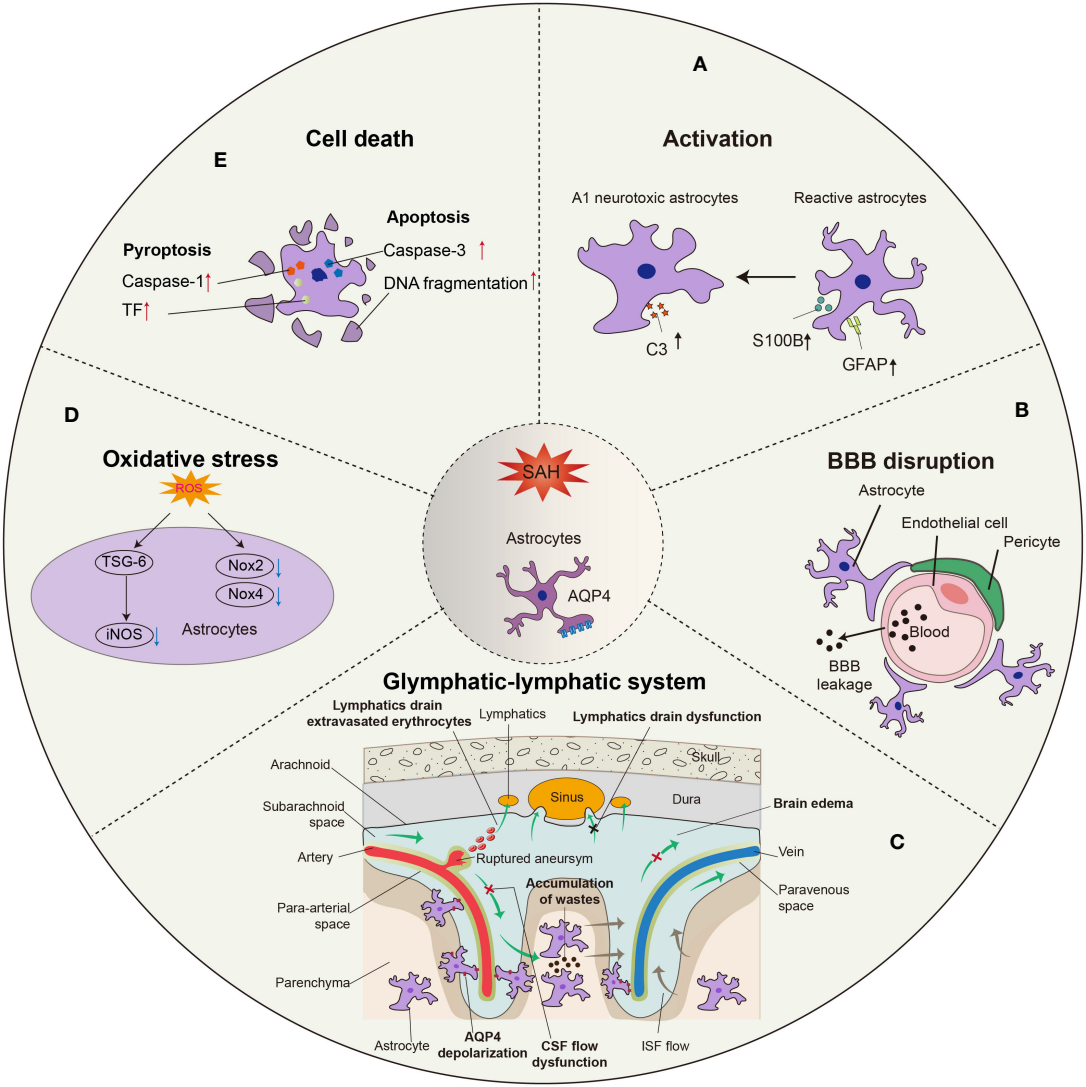
Morphological and functional changes in astrocytes after SAH. **(A)** Astrocytes are activated into predominant neurotoxic A1 subtype after SAH. **(B)** Astrocytes are involved in the disruption of BBB integrity due to SAH, leading to the leakage of circulatory neurotoxic substances into the parenchyma. **(C)** Astrocytes-constituted glymphatic-lymphatic system is impaired due to the hemorrhage in subarachnoid space, resulting in subsequent brain edema. The polarity of AQP4 in astrocytic endfeet is decreased after SAH. **(D)** Excess ROS in astrocytes is related to the oxidative stress after SAH, in which iNOS, Nox2, and Nox4 play critical roles. **(E)** The apoptosis of astrocytes occurs earlier than neurons after SAH, probably in a caspase-dependent manner.

During SAH, astrocytes become activated, which includes upregulation of glial fibrillary acidic protein (GFAP) and S100 calcium binding protein B (S100B). Rather than the A2 phenotype, astrocytes preferentially take on the A1 phenotype, which plays a harmful role in the pathophysiological process after SAH. In addition, astrocytes participate in BBB disruption and glymphatic-meningeal lymphatic system dysfunction *via* aquaporin-4 (AQP4) depolarization (**Figure 1**). As an essential part of the neurovascular unit (NVU), interactions between astrocytes and other cells, such as neurons, endothelium, pericytes, and microglia, are involved in SAH.

### Astrocyte activation and phenotypic changes

Astrocytes are activated when the brain sustains an injury. The process of astrocyte activation, known as ‘reactive gliosis,’ is a reaction that includes changes in structure and function. Upregulation of GFAP in astrocytes is perhaps the most common hallmark of reactive astrocytes and reactive gliosis (10). Under pathological conditions, activated astrocytes show two different histological phenotypes sequentially, namely, reactive astrocytes (RAs) and scar-forming astrocytes (SAs). Increased GFAP expression and morphological changes are thought to be the minimum standard for defining RAs. Furthermore, S100B, which is mainly expressed in astrocytes, is known as a reliable biomarker of neural diseases (11). In SAH patients, GFAP and S100B were elevated in both CSF and serum (12–14), and S100B is known as an important biomarker for predicting the prognosis of SAH (15, 16). In recent clinical research, GFAP and S100B levels were increased in SAH patients at the time of SAH diagnosis and 24 h after the event. Both biomarkers were associated with increased mortality (17). In animal experiments, GFAP levels increased after SAH and then gradually recovered to baseline (18–20). Moreover, there is a significant increase in S100B in SAH-induced vasospasm (21). The evidence indicated the existence of reactive astrogliosis after SAH. Whether astrocyte activation is detrimental or beneficial to the recovery of SAH remains controversial, as activation of astrocytes is categorized as neurotoxic A1 or neuroprotective A2 (22). Through transcriptomic methods, Zamanian et al. found that neuroinflammation and ischemia induced two different types of reactive astrocytes, termed A1 and A2, respectively. H2-D1, C3 and serping1 are markers for the A1 subtype, and pentraxin 3 (PTX3), sphingosine-1-phosphate receptor 3 (S1PR3) and S100 calcium-binding protein A10 (S100A10) are markers for the A2 subtype of reactive astrocytes (23). Recent research has shown that C3 and serping1 immunoreactivity are the common markers that represent the A1 phenotype, while S100A10 and PTX3 are the common markers of A2 astrocytes (24–28). Animal research showed that astrocytes were transformed into A1 astrocytes, and A1 astrocytes caused cell death by releasing proinflammatory factors after SAH (5). Ma et al. demonstrated that both A1 and A2 astrocytes existed after SAH in patients’ brain tissue, but A1 was predominant and more sustained than A2. Furthermore, they indicated that the expression of C3 is more increased than that of PTX3 in an SAH rat model. These results showed that A1-type astrocytes were more predominant than A2-type astrocytes in both SAH patients and animal SAH models (4). Thus, novel methods for regulating astrocytic phenotypes might provide new therapeutic benefits. However, there appear to be discrepancies between some of the reported observations. With the development of genomic sequencing methods and acknowledgment of the pathological role that astrocytes play in neurological diseases, the phenotype of astrocytes is increasingly regarded as a spectrum ranging from pro- to anti-inflammatory according to their differences in gene expression instead of simply binary classification to A1 and A2 (29). Therefore, a precise classification of astrocytes is needed to develop astrocyte-targeted therapy after SAH.

### Astrocytes and BBB disruption

The BBB is mainly composed of tightly sealed endothelial cells, tight junction proteins, basal lamina, pericytes, and astrocytic endfeet (30). Astrocytic endfeet surround cerebral vessels and thus participate in the formation of the BBB. The BBB is critical for proper neuronal functions, such as preventing toxins and pathogens from entering the brain, maintaining homeostasis in the CNS, and preventing neurotransmitters from entering the blood (31).

The earliest observation of BBB changes after SAH began in 1983. Peterson and Cardoso established an experimental SAH model in cats by subarachnoid blood injection and measured BBB function by Even’s Blue. According to their description, ‘SAH does not cause BBB breakdown to proteins during its acute stage’ (32). In addition, a clinical study reported that only 1 out of 26 SAH patients showed a BBB disruption, as detected by positron emission tomography with 68-Ga-EDTA (33). Those conclusions that BBB is not or merely slightly impaired after SAH are perhaps due to the limitations of observation methods decades ago. In 1992, Germanó et al. summarized previous investigations concerning the occurrence of BBB breakdown after experimental SAH and assessed BBB changes utilizing the newly developed quantitative technique (34). According to their study, the ability of radiolabeled alpha-aminoisobutyric acid to cross the BBB was 1.3-1.5 times greater in the SAH group than in the sham group, suggesting BBB breakdown after SAH. Since then, with the development of various detection technologies, numerous studies over the past decades have established that BBB permeability is indeed disrupted after SAH (35–37).

Several studies, including our previous work, have demonstrated that matrix metalloproteinase-9 (MMP-9) leads to the disruption of the BBB through the degradation of basal lamina proteins, tight junctions, and the extracellular matrix in neurologic diseases, such as cerebral hemorrhage (38–40). MMP-9 is a member of the zinc- and calcium-dependent proteolytic enzyme family and is widely distributed in multiple cerebral cell types, including astrocytes (41). Herein, we review whether MMP-9 expressed in astrocytes plays a role in the pathogenesis of BBB disruption after SAH. Cao et al. reported that BBB disruption after SAH was related to MMP-9-induced damage to tight junction proteins and was ameliorated by hydrogen sulfide (42). In addition, Okada et al. confirmed that secreted protein acidic and rich in cysteine (SPARC), a type of matricellular protein, was secondarily increased in astrocytes in response to injury after SAH and led to BBB breakdown, possibly mediated *via* the mitogen-activated protein kinases (MAPKs)/MMP-9 signaling pathway (43). In SAH patients, Tenascin-C is increased in astrocytes following SAH and may be involved in BBB disruption, neuroinflammation and cerebral vasospasm in EBI (44). In addition to the above, astrocyte-derived neurotrophic factors are also important. Secretion of astrocyte-derived neurotrophic factors is increased after SAH, and injection of exogenous neurotrophic factors abates BBB damage *via* by suppressing MMP-9, suggesting a significant neuroprotective role of neurotrophic factors derived from astrocytes (8).

In addition to the mechanisms mentioned above, astrocytes also exert effects on the BBB in an MMP-9-independent manner after SAH. Suzuki et al. revealed that osteopontin (OPN) expressed by astrocytes was increased after SAH (45). BBB disruption was exacerbated by inhibiting OPN expression and was ameliorated by recombinant osteopontin (r-OPN). In addition, BBB disruption was also related to iron overload, in which ferritin expressed in different types of cells, including astrocytes, was demonstrated to be essential (46). Furthermore, AQP4 is also closely related to the function of BBB. The glial membrane is studded with orthogonal arrays of intramembranous particles (OAPs). Among them are AQP4 and the dystrophin-dystroglycan complex (DDC) important for BBB integrity. It has been reported that there existed a positive relationship between OAPs-based AQP4 and the function of BBB, suggesting that AQP4 played a critical role in the BBB maintenance (47). In the studies of SAH mice, the up-regulation of AQP4 ameliorated the BBB injury and effectively improved the neurobehavioral ability (48), while the down-regulation of AQP4 has no effect on BBB permeability (49). AQP4 may influence the BBB function by regulating the expressions of laminin and tight junction proteins, which has been proved to be critical components of BBB structure (50). 

Regarding neuroinflammation, astrocytes are reportedly integral to BBB breakdown. For instance, Angiogenic factor with G patch and FHA domains 1 (Aggf1), initially identified as a vascular endothelium-derived protein, was expressed in astrocytes and was obviously increased over time in rats after SAH (51). Knockdown of endogenous Aggf1 exacerbated BBB breakdown, whereas exogenous Aggf1 improved BBB integrity and long-term neurological scores after SAH, suggesting that Aggf1 protects the integrity of the BBB. Intriguingly, exogenous Aggf1 also exerts anti-inflammatory effects by inhibiting the activation of microglia and astrocytes, thus contributing to a better outcome after SAH. Such results and other evidence indicate the occurrence of neuroinflammation during BBB destruction (52–54). On the one hand, neuroinflammation causes BBB disruption. On the other hand, leakage of the BBB enables the entrance of toxins and pathogens, which exacerbates neuroinflammation and eventually closes a negative feedback loop after SAH.

### Astrocytes and glymphatic-meningeal lymphatic vessel drainage system

The classic model of CSF circulation theory acknowledges that cerebrospinal fluid (CSF) is produced by the choroid plexus, circulated into the subarachnoid space, and absorbed through arachnoid villi (55). Any obstruction in this circulation may consequently lead to the accumulation of CSF in communicating hydrocephalus (55). However, a cerebral waste clearance system was discovered by Iliff et al. in 2012 (56). Through the application of fluorescent tracers injected into the cisterna magna and circulated along with CSF, the dynamics of the “glymphatic system” were characterized for the first time under a two-photon microscope. CSF enters the perivascular spaces of the penetrating arteries, which are formed by astrocytic endfeet. By the transporting action of AQP4 protein expressed in astrocytic endfeet, CSF then enters the parenchyma and forms bulk flow. The waste products in interstitial fluid (ISF), such as β-amyloid, are removed by the CSF bulk-flow. CSF enters the perivenous space, further drains out of the brain *via* olfactory-nasal lymphatics and newly discovered meningeal lymphatic vessels, and eventually into the deep cervical lymph nodes (dCLN) (57). Further studies reveal that the main driving force of this system is the pulsation of the penetrating artery (58). Moreover, the function of this cerebral lymphatic drainage system is impaired in a wide range of neurological diseases, such as ischemic stroke, cerebral hemorrhage, and traumatic brain injury (59).

Glymphatic system and meningeal lymphatic vessel dysfunction may contribute to the development of hydrocephalus (60), which is responsible for prolonged hospitalization and poor outcomes after SAH (61). Several studies have independently demonstrated that glymphatic influx function and meningeal lymphatic drainage are severely impaired after SAH both in murines and nonhuman primates (62, 63), suggesting that disorders of this newly identified CSF pathway may also participate in the pathogenesis of SAH, in addition to classic CSF circulation theory. However, the concrete role of the glymphatic system in SAH remains unknown. One interesting study conducted by Golanov et al. revealed that tissue factors secreted by astrocytes in the glymphatic system may regulate CSF flow and localize hemorrhage by reducing the deposition of fibrin in SAH (64). In a series of studies, the meningeal lymphatic drainage pathway was surgically blocked *via* ligation of the dCLN, and it was found that such blockade leads to worse brain edema and OS following the acute stage of SAH (65, 66). In addition, meningeal lymphatics are also reported to drain extravasated erythrocytes from CSF into CLNs after SAH, suggesting that modulation of meningeal lymphatic vessels may be a promising approach to ameliorate SAH outcomes (67). Even so, further preclinical and clinical studies are still needed to explore the possibility of the impaired glymphatic and its drainage system as a potential therapeutic target in SAH.

The AQP family has long been regarded as a privileged target for edema modulation. Among them is AQP4, the best-characterized AQP, and the AQP that has the greatest impact on water movement (68). Previous studies have indicated that AQP4 is involved in the development of brain edema. The main types of brain edema are cytotoxic, vasogenic, and hydrocephalic. Mounting evidence demonstrated that AQP4 is reported to accelerate the formation of cytotoxic brain edema, including intracellular accumulation of water in neurons and astrocytes (69). For example, in a mouse middle cerebral artery occlusion (MCAO) model study, AQP4-null mice show a decreased edema volume, neuron death, and neuroinflammation from 3 days to 14 days after the insult (70). In addition, reduced brain swelling after brain ischemia has also been reported in α-syntrophin-null mice (71), suggesting that AQP4 expressed on the astrocytic endfeet may play an important role in cytotoxic edema. In contrast, AQP4 served a protective role in vasogenic brain edema, which terms the water accumulation in the extracellular space caused by the disruption of BBB integrity (69). Transcellular water movement mediated by AQP4 is proved to be crucial for fluid clearance in vasogenic brain edema in several mouse disease models such as freeze-injury and traumatic brain injury (72, 73). 

AQP4 is also a key protein that constitutes the glymphatic system and plays a central role in transporting CSF across the astrocytic endfeet into the brain parenchyma. Therefore, when the glymphatic system is mentioned, AQP4 changes after SAH are an interesting topic always worth discussing. A series of recent studies have indicated that the total amount of AQP4 is significantly increased after SAH and decreased with improved outcomes after early interventions such as hydrogen sulfide (42, 74, 75). These imply that AQP4 may play a deleterious role during the development of brain edema after SAH. 

Interestingly, it has been reported that the length density of AQP4-positive capillaries in the hippocampus is lower in an SAH model than in a sham control, which seems to be inconsistent with the above conclusions (76). In addition, Liu et al. applied a combination of magnetic resonance imaging (MRI), confocal microscopy, and transmission electron microscopy, demonstrating that the function of the glymphatic system is impaired after SAH and even worse in AQP4-null mice (77). Mice without AQP4 show worse performance in neurologic scores and develop more severe brain edema by measuring brain-water content. This evidence suggests that AQP4 may also have protective effects on outcomes after SAH to some degree. Such a ‘double-edged sword’ effect of AQP4-change in the pathophysiology after SAH warrants further studies. One possible explanation for the dual effects of AQP4 change is that AQP4 changes both in total amount and in polarity. The polarization of AQP4 is essential for the proper function of the glymphatic system, and SAH leads to a depolarization of AQP4 according to a previous study (63). The increased expression of AQP4 could be the result of astrocytic activation due to SAH, and most of them are expressed in the soma rather than the endfeet of astrocytes, which consequently leads to the depolarization of AQP4, resulting in the dysfunction of the glymphatic system. Intriguingly, there also exists a controversial conclusion that AQP4-null mice significantly reduce the movement of blood into the parenchyma but alleviate neither neuroinflammation nor neurological deficits after SAH, suggesting that vasculitis and neuroinflammation after SAH are independent of glymphatic control (78).

### Astrocytes and OS

OS refers to a state of imbalance between oxidation and antioxidant processes in the body. Overproduction of reactive oxygen species (ROS) and diminished antioxidant capacity in the body result in excess ROS, which induces cell damage. EBI after SAH covers a series of important pathological changes in the brain from the initial hemorrhage to the occurrence of vasospasm within 72 h (79).

OS plays an important role in pathological changes in EBI, and increasing evidence indicates that antioxidative therapy is an effective method for EBI. After SAH, extracellular free oxyhemoglobin is the main source of free radicals. Upregulation of inducible nitric oxide synthase (iNOS) and NADPH oxidase (NOX), as well as inhibition of endogenous antioxidant systems, such as superoxide dismutase (SOD) and glutathione peroxidase (GSH-Px), also play an important role in this pathological change. iNOS is overexpressed in astrocytes under some pathological conditions. A previous study showed that tumor necrosis factor-α stimulated gene-6 (TSG-6) could downregulate the expression of iNOS in astrocytes after SAH (80). In an animal experiment, Nox2 and Nox4 both had a significant increase in astrocytes at 12 hours after SAH, which generated ROS in astrocytes after SAH by transferring electrons from NADPH to oxygen molecules (81). The expression of glutathione peroxidase-4 (GPx4) is increased in reactive astrocytes after cerebral injury (82). Whether the expression of SOD and GSH-Px increases after SAH, similar to other cerebral injuries, is understudied. In addition, recent evidence has suggested that regulating nuclear factor erythroid 2 (NF-E2)-related factor 2 (Nrf2) offers a possible approach to SAH therapy. Kelch-like ECH-associated protein 1 (Keap1) is bound to Nrf2 in the cytoplasm under normal circumstances. When Keap1 detects the occurrence of OS, Nrf2 binds to the antioxidant response element (ARE), regulating the transcription of a series of detoxifying or antioxidant enzymes (83). The absence of Nrf2 will cause more injury *in vitro* and *in vivo*. In an *in vitro* SAH model, astrocytes from wild-type or Nrf2 knockout mice were exposed to oxyhemoglobin. The study indicated that the transcription factor nuclear factor-κB (NF-κB) was upregulated in astrocytes after oxyhemoglobin treatment. Meanwhile, the expression of inflammatory cytokines, including tumor necrosis factor α (TNF-α), interleukin-1β (IL-1β), interleukin-6, and MMP9, increased. Depletion of Nrf2 aggravated the inflammation mentioned above. A series of recent studies have indicated that Nrf2 depletion accentuates astrocyte damage following SAH, whereas Nrf2 activation reduces deficits. To date, however, there has been very little discussion regarding human data; this deficiently urgently needs to be addressed.

### Cell death in astrocytes

Previous research has established that cell apoptosis might play an important role in brain injury after SAH. Apoptotic cell death occurs very early after SAH. As a result, there are potential therapeutic targets to intervene during apoptosis that may ameliorate brain injury following SAH. The mechanisms involved in apoptosis after SAH include intrinsic and extrinsic pathways. The intrinsic pathways, also known as mitochondrial pathways, include caspase-dependent and caspase-independent pathways (84). Caspase-9 is the initiating factor of cascades, and it activates caspase-3, which is considered to be one of the final steps of apoptosis. Activated caspase-3 can shear several key cellular proteins and promote DNA fragmentation and nuclear decomposition, which ensure the completion of energy-dependent apoptosis (85). In a dog SAH model, colocalization of caspase-3 with GFAP increased in the hippocampus, cortex, and brain stem compared with the control group, indicating apoptotic astrocytes (86). Similarly, in a rat model, there was a significant increase in TUNEL-positive cells after SAH. Approximately 20% of the TUNEL-positive cells were astrocytes, which means astrocytic apoptosis was involved in SAH. DNA fragmentation was observed in TUNEL-positive cell areas, which are in proximity to the injection site. Furthermore, the acute decrease in cerebral blood flow was related to the degree of cell apoptosis. Furthermore, more cells labeled with GFAP showing DNA fragmentation were observed 2 days after SAH than 7 days after SAH, which suggests that degeneration of astrocytes may have occurred earlier (87). Interestingly, these results indicated that glial cells might be more susceptible to SAH than neurons. However, it is conventional wisdom that neurons are the most susceptible cells to brain injury, which indicates that there might be some different mechanisms between SAH and other cerebral diseases.

Pyroptosis is a type of caspase‐1-dependent cell death that involves the canonical and noncanonical pathways. In the canonical pathway, inflammasomes activate caspase-1, which induces lytic cell death through the effector protein gasdermin D (GSDMD) (88). Previous research showed that the expression levels of caspase-1 and tissue factor (TF) in astrocytes were significantly increased from 1 h to 3 d after SAH *in vivo*. Moreover, pyroptotic cells and TF were further increased in primary astrocytes after hemoglobin, while inhibition of caspase-1 successfully attenuated TF and inflammatory cytokine release *via* GSDMD cleavage-mediated membrane pore formation in astrocytes. Thus, activated caspase-1 induces the astrocytic release of TF through pyroptosis after SAH (89). Furthermore, Gu et al. indicated that CXCL-12 induced neuronal pyroptosis *via* the CXCR4/NLRP1 signaling pathway after SAH. Immunofluorescence staining showed that CXCR4 was also expressed on astrocytes, which means that astrocytes might be involved in neuronal pyroptosis in EBI after SAH (90).

Ferroptosis is a new form of iron-dependent programmed cell death. Previous studies have shown that neuronal ferroptosis occurs in SAH and that the infiltration of transferrin into the brain parenchyma might play an important role in neuronal ferroptosis after SAH (48, 91, 92). Liu et al. demonstrated that AQP4, which is located at the astrocyte foot, would reduce the transferrin content and neuronal ferroptosis in SAH mice (48). In conclusion, there is a limitation of the research about pyroptosis and ferroptosis in astrocytes after SAH, although these two types of cell death play a critical role in SAH.

## Alterative crosstalk between astrocytes and other cerebral cells after SAH

### Neuron

Some evidence has shown that the progression of SAH is related to the degree of neuronal death. Controlled neuronal death may attenuate EBI after SAH. Caspase activation peaked in neurons at 24 h after SAH (93). Modulation of the sphingosine-1-phosphate receptor on astrocytes can mediate A1 astrocyte activity, reduce neuronal death, attenuate neuronal death, and improve neurobehavioral functions in rats subjected to SAH (5). Mesencephalic astrocyte-derived neurotrophic factor (MANF), a secreted neurotrophic factor, has neuroprotective effects on neurons by relieving ER stress after SAH (8). Meanwhile, the secretion of prokineticin 2 (PK2) in neurons promoted by TNF-α can induce the differentiation of astrocytes from an A1 phenotype into an A2 phenotype through the activation of signal transducer and activator of transcription 3 (STAT3) (4). Recent research showed that the expression of CD24 increased in hippocampal astrocytes after SAH *in vitro*. Moreover, they also indicated that downregulation of CD24 in astrocytes resulted in aggravated neuronal apoptosis, in which the SHP2-ERK signaling pathway and the brain-derived neurotrophic factor (BDNF) might be involved (94).

### Endothelial cells

Brain endothelial cells (ECs) comprise the majority of the NVU, but aneurysmal rupture and SAH induce EC dysfunction and apoptosis (95). Iron overload and subsequent OS exerted cytotoxic effects on brain ECs, activating apoptotic cell death. Prior evidence showed that caspase-3 activation and DNA fragmentation started within ten minutes in endothelial cells after SAH (6, 95). Blood components in the subarachnoid space, including thrombin, heme, fibrinogen, and leukocytes, can also activate Toll-like receptor 4, which produces proinflammatory mediators, such as MMP-9, IL-1β, and TNF-α, causing EC dysfunction and the migration of macrophages into the subarachnoid space (96). Activated astrocytes synthesize excitatory transmitters, such as glutamate, which activate N-methyl D-aspartate receptors (NMDARs) and cause excessive Ca^2+^ influx and have a toxic effect on ECs (6). Anthony C Pappas et al. demonstrated that the amplitude of spontaneous Ca^2+^ increased in astrocyte endfeet, and NVC changed from vasodilation to vasoconstriction in SAH model rats (97). Meanwhile, astrocytes secrete a range of factors, including glial-derived neurotrophic factor (GDNF), transforming growth factor-β (TGF-β), and basic fibroblast growth factor (bFGF), which enhance the barrier function of ECs *in vitro* (98). In addition, endothelial cells also affect astrocytes after SAH. Evidence shows that homodimers of platelet-derived growth factor-β (PDGF-β) derived from endothelial cells and pericytes, which are stimulated by degradation products of blood in the perivascular space after SAH, activate astrocytic platelet-derived growth factor receptor-β (PDGFR-β) signaling and promote local tissue responses and functional recovery (99).

### Microglia

As the resident immune cells in the CNS, microglia and astrocytes communicate and cooperate in the brain and take the shape of a cascade immune network of amplification to stimuli (100). Here, we focus primarily on the crosstalk between microglia and astrocytes after insult. In the acute phase, microglia are more sensitive to stimuli and are first activated into the proinflammatory M1 phenotype. Activated microglia secrete cytokines and chemokines to trigger reactive astrocytes, such as IL-1β, TNF-α, and HMGB1 (18, 51, 101, 102). In turn, proliferated and activated astrocytes also participate in the regulation of the activation of microglia, possibly *via* the release of molecular signals and the action of cellular connexin channels (90, 100). At the chronic injury stage, microglia and astrocytes undergo a phenotypic transition from the proinflammatory M1 and A1 subtypes to the anti-inflammatory M2 and A2 subtypes. M2 microglia are characterized by cytokines, including IL-4 and TGF-β (103). A study revealed that regulating microglial phenotype polarization to the M2 phenotype through the P38/STAT6 pathway improves the outcome after SAH, suggesting the essential role that M2 microglia play in recovery following SAH (104). A2 astrocytes reportedly participate in the regulation of M2 microglial activation *via* TGFβ/IL-10 signaling under LPS stimulation (7). Whether it also occurs in the chronic stage after SAH and whether there exists other possible M2-A2 crosstalk remain to be explicated. In addition, as mentioned above, the traditional binary pro- and anti-inflammatory classification of glial cells cannot cover all cellular phenotypes under disease conditions. Therefore, it is meaningful to explicate spectrum changes of microglia and astrocytes and their communications after SAH, which may rely on the wide applications of the cellular sequencing technique.

### Pericytes

Pericytes play an important role in vasoconstriction, angiogenesis, and immune or phagocytic function. The signaling communications of pericytes with astrocytes include the CypA-NFκB-MMP-9 pathway, arachidonic acid pathway, and Ca^2+^-calmodulin-myosin light chain (MLC) pathway (105). In addition to the MMP-9 produced by astrocytes mentioned above, pericytes are believed to secrete MMP-9 in an astrocyte-dependent manner *via* the CypA-NFκB pathway, which further exacerbates the breakdown of the BBB structure in SAH (9). Delayed cerebral ischemia after SAH results in severe functional and cognitive deficits, which are due to vasospasm caused mainly by the abnormal constriction of pericytes (106, 107). Although there are studies concerning Ca^2+^-related pericyte constrictions in other neurological disorders, such as ischemic stroke (108, 109), evidence regarding how astrocytes regulate pericyte constrictions following SAH is still insufficient and thus remains to be further explored.

### Other cells

In addition, previous research showed that a large number of monocytes infiltrated the brain after SAH (110). Furthermore, Gris et al. indicated that macrophage activation, as well as neutrophil and classical monocyte infiltration, occurred after SAH (111). There is a lack of the research about interactions between other cerebral cells such as oligodendrocyte, oligodendrocyte precursor cells and astrocytes after SAH, which may be a research priority in the future. 

## Neuroprotective functions of astrocytes in SAH

**Figure 2:**
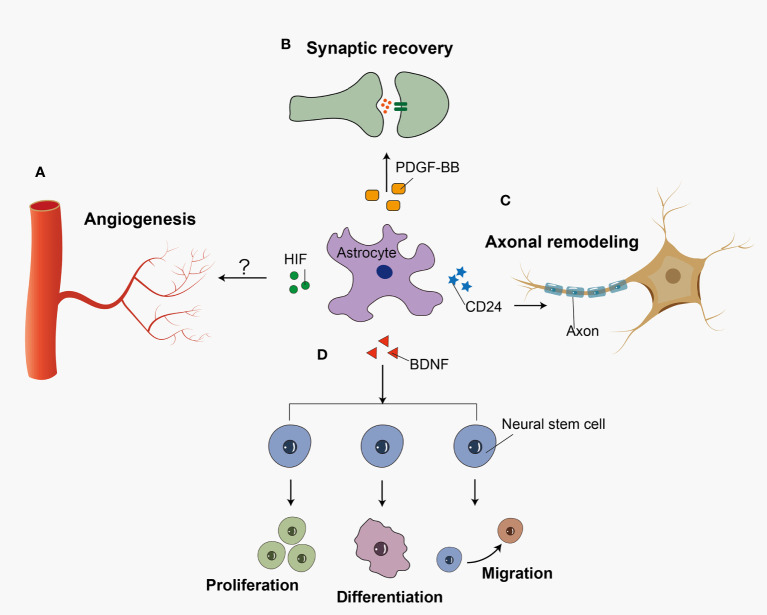
Reactive astrocytes play a neuroprotective role in recovery stage after SAH. Reactive astrocytes release several cell cytokines, playing a neuroprotective role after SAH. **(A)** HIF accelerates the angiogenesis possibly *via* WNT signaling pathway. **(B)** PDGF-BB facilitates the recovery of synapses between neural cells. **(C)** CD24 participates in the axon regeneration after the insult. **(D)** And BDNF promotes the proliferation, differentiation, and migration of neural stem cells.

Reactive astrocytes exert many neuroprotective functions including angiogenesis, synaptogenesis, axonal remodeling, and neurogenesis in SAH (**Figure 2**). A recent study showed that hypoxia-induce factor (HIF) increased in an SAH mouse model (112). In astrocytes, HIF regulates WNT signaling to generate new blood vessels (113). Astrocytes may participant in the angiogenesis after SAH, which has not been studied yet. Previous research demonstrated that astrocytes played an important role in synaptic recovery through PDGF-BB signaling after SAH (99). Zuo et al. demonstrated that 5-Bromo-2’-deoxyuridine (BrdU) positive cells were co-labeled with GFAP in astroglial scar at 14 days after SAH. In the recovery stage of SAH, majority of BrdU positive progenitor cells differentiate into neurons rather than activated astrocytes after SAH (19). Following SAH, BDNF expression was upregulated in astrocytes, which promoted the proliferation, differentiation, and migration of neural stem cells (114). In contrast with the traditional viewpoint, Anderson et al. indicated that astrocyte scar formation helped rather than prevented CNS axon regeneration (115). However, overactivation of astrocytes leads to glial scar formation and inhibits axonal regeneration. Previous *in vitro* research showed that the knock-down of CD24 in astrocytes leaded to the impairment of the axon in SAH (116). Because so little work has been done in this area, it is difficult to make any firm conclusions about what role astrocyte-driven axonal remodeling has in SAH. 

## Astrocytes as a new therapeutic target in SAH

Astrocytes play a dual role in cerebral injuries, and determining how to improve their helpful effects and/or reduce their detrimental effects is a crucial challenge. We therefore summarize reported treatments that target astrocytes for SAH treatment. (**Table 1**). At present, most research regards A1 as representative of neurotoxic astrocytes and A2 as representative of neuroprotective astrocytes. Therefore, attempting to suppress the activation of A1 may be a new therapeutic strategy for SAH. Ponesimod is an oral drug approved for the treatment of adults with relapsing multiple sclerosis. It may help reduce the migration of lymphocytes to the CNS, which could reduce inflammation during disease. A potential neuroprotective agent regarding astrocyte phenotypes might be ponesimod, which displayed neuroprotective effects by preventing astrocytes from transferring to the A1 phenotype after SAH and was regulated by STAT3 signaling. Ponesimod attenuated SAH-induced inflammation, neuronal apoptosis, and neurological deficits (5). In addition, TNF-α helped the transformation of A1 to A2 by upregulating the expression and secretion of PK2 in neurons. Although its protective roles are minimal, PK2 induced by TNF-α might be an endogenous mechanism for self-repair after SAH (4). A potential neuroprotective agent might be gastrodin. SAH-induced astrocyte activation could be significantly reduced by gastrodin, as gastrodin attenuated neurological damage and brain edema following SAH (117). Collectively, selectively modulating astrocytes into a protective phenotype might be a useful therapy for SAH.

**Table 1:** Treatments targeting astrocytes for SAH, their potential mechanisms, and curative effects.

Target	Treatment	Model	Mechanism	Curative effect	Reference
Inhibit excessive activation of astrocytes	ponesimod	*In vivo*	Suppress the activation of A1 astrocytes through STAT3 signaling	Inhibit inflammatory response and reduce neuronal death	(5)
	PK 2	*In vivo*	*Promote the formation of A2 reactive astrocytes through the STAT3 pathway*	*Attenuate neuronal damage and behavioral dysfunction*	(4)
	Gastrodin	*In vivo*	Reduce the astrocyte activation	Attenuate neurological damage and brain edema	(117)
Maintain the integrity of BBB	TSG-6	*In vivo In vitro*	Downregulate the expression of iNOS and the NF-κB/MAPK pathway in astrocytes	Protect the BBB function	(80)
	MANF	*In vivo In vitro*	suppress the expression of MMP-9 and caspase-3	Improve neuro-functions	(8)
	Rh-Aggf1	*In vivo*	Provide neuroprotection *via* PI3K/Akt signaling pathway	Reduce BBB disruption, neuroinflammation, and consequent brain edema	(51)
	R-OPN	*In vivo*	Increase MKP-1 and downregulate the VEGF-A	Reduce BBB disruption	(43)
Improve glymphatic system function	Nimodipine	*In vivo*	Improve the glymphatic system function by alleviating astrocytes depolarization	Reduce brain edema and attenuate neurologic dysfunction	(118)
	PACAP	*In vivo*	Attenuate the glymphatic system dysfunction	Reduce brain edema and BBB damage. improve neurological deficits	(61)
	Atorvastatin	*In vivo*	Reduce the expression of AQP4 and ER stress	Reduce brain edema and cell apoptosis. Glymphatic not evaluated	(119)
Regulate NVU	VX-765	*In vitro and in vivo*	Inhibit the inflammasome activation	Reduced astrocytic pyroptosis and neuron loss	(28)
	Aggf1	*In vivo*	Inhibit the activation of glia cells by reducing the expression of TNF-α and IL-1β	Attenuate the neuroinflammation after SAH	(51)

(PK2, prokineticin 2; TSG-6, tumor necrosis factor-α stimulated gene 6; BBB, blood-brain barrier; MANF, mesencephalic astrocyte-derived neurotrophic factor; MMP-9, matrix metallopeptidase-9; Rh-Aggf1, recombinant human angiogenic factor with G patch and FHA domains 1; R-OPN, recombinant osteopontin; VEGF-A, vascular endothelial growth factor A; PACAP, pituitary adenylated cyclase-activating polypeptide; ER, endoplasmic reticulum; NVU, neurovascular unit; TNF-α, tumor necrosis factor-α; IL-1β, interleukin-1β; SAH, subarachnoid hemorrhage).

Astrocytes play an essential role in maintaining the integrity of the BBB. Neuroinflammation and OS are involved in BBB disruption following SAH. TSG-6 is secreted by bone marrow mesenchymal stem cells (BMSCs), which protect the BBB by downregulating the expression of iNOS and inhibiting the NF-κB/MAPK signaling pathway in astrocytes. Silencing TSG-6 in BMSCs aggravated the damage to astrocytes and the BBB (80). However, the existing accounts fail to resolve the mechanism behind this observation. MANF was first found in a rat mesencephalic astrocyte cell line, and MANF treatment improved neurofunctions in SAH animals by cleaving caspase-3 and suppressing the expression of MMP-9 (8). Zhu et al. demonstrated that recombinant human Aggf1 (rh-Aggf1) treatment rescued the disruption of the BBB and attenuated neuroinflammation after SAH, which was reversed when Aggf1 was knocked down, and rh-Aggf1 played a neuroprotective role *via* the PI3K/Akt/NF-κB pathway (51). OPN was expressed in reactive astrocytes during EBI in a rat SAH model. R-OPN treatment reduced BBB damage at 24 h after SAH. Upregulation of the mitogen-activated protein kinase phosphatase-1 and downregulation of vascular endothelial growth factor-A *via* OPN after SAH might be the protective mechanism of this treatment (45). Importantly, however, inflammation is a double-edged sword. As mentioned above, most interventions are anti-inflammatory. However, the protective effect of inflammation is often overlooked.

As mentioned above, the total amount of AQP4 is increased and the polarity of AQP4 is decreased after SAH. Considering AQP4 plays a critical role in SAH, it may be promising to take astrocytic AQP4 as a therapeutic target. Previous studies focused on rescuing glymphatic dysfunctions by regulating AQP4. For example, nimodipine is traditionally used in clinic to alleviate vasospasm following SAH *via* calcium antagonism (120). Nevertheless, recent evidence suggests that nimodipine improves glymphatic system drainage in SAH and reduces the depolarization of astrocytes while improving the expression of AQP4 *via* activation of the cAMP/PKA pathway. In this way, nimodipine played a neuroprotective role in alleviating neurological deficits and brain edema by improving the function of the glymphatic system (118). Accordingly, damage to the glymphatic system would result in the accumulation of metabolic toxins within the brain parenchyma, possibly leading to aggravated neurological dysfunction and cognitive deficits after SAH. on this basis, treatments aiming AQP4 polarity to improve the glymphatic system might be able to reduce deficits following SAH.

In addition to glymphatic modulating, AQP4-based therapeutic strategies may be involved in the formation of brain edema and maintenance of BBB integrity. Atorvastatin significantly reduced the expression of AQP4 and brain water content after SAH, although the role of the glymphatic system is less understood (119). Hydrogen sulfide attenuated brain edema in SAH rats by inhibiting BBB breakdown *via* reducing MMP-9 and AQP4 expression (42). Despite all these endeavors, there are still lacking specific methods to singly modulating AQP4. Previous approaches of regulating AQP4 in the CNS included gene silencing or activation by microRNAs, protein modification such as phosphorylation, and direct inhibition of ions or compounds, TGN-020, for example (121). Up to now, those interventions have not proved to be effective in SAH modeling mice. There may be still a long way to go before targeting AQP4 become a reliable therapeutic strategy. 

Astrocytes make dual and central contributions to the integration of neurons and vasculature. Regulating astrocytes with other cerebral cells may be a new strategy for SAH. For example, inhibition of caspase-1-mediated inflammasome activation *via* VX-765 reduced astrocytic pyroptosis in an *in vitro* SAH model and prevented hydrocephalus and hippocampal neuron loss in a rat model of SAH (89). Research has indicated that regulating inflammation after SAH can coordinate functions among astrocytes and neurons. In addition, some interventions reportedly have an anti-inflammatory function since they can suppress communication between astrocytes and microglia. For example, exogenous Aggf1 reduces the release of molecular signals, such as TNF-α and IL-1β, thus inhibiting the activation of glial cells and attenuating neuroinflammation after SAH (51). In addition, it has been reported that SAH-induced changes in astrocyte Ca^2+^ signaling lead to a switch in the polarity of the neurovascular coupling response from vasodilation to vasoconstriction (97). Therefore, whether reducing astrocyte Ca^2+^ could change vasoconstriction and increase blood flow after SAH may be a research priority.

## Conclusion

Despite decades of exploration, the prognosis following SAH remains far from satisfactory. The pathological mechanisms of SAH are complex. Astrocytes are among the key components of the CNS, and they appear to play an important role in the pathogenesis and prognosis of SAH. In this review, we summarize how astrocytes become activated and are involved in OS, cell death, BBB disruption, and the glymphatic-meningeal lymphatic system during SAH. In addition, astrocytes play a neuroprotective role in recovery stage of SAH. Some potential agents targeting astrocytes have been studied in SAH. However, because there is much less information about SAH from human research than from model systems, there is a clear need for clinical studies to examine the safety and efficacy of astrocyte-targeted treatment after SAH. Determining how to translate research results from animal studies to humans has become a crucial problem.

## Author contributions

RL, MZ, and YH conceived the main outline. RL and MZ wrote the manuscript. DY, RL, and XZ made the figures. LW, CL, and YO took charge of manuscript revision in English. YH participated in the correction and finally reviewed this article. All authors contributed to the article and approved the submitted version.

## Acknowledgments

We would like to acknowledge the help of all the staff involved in taking part in the paper editing and literature review.

## Conflict of interest

The authors declare that the research was conducted in the absence of any commercial or financial relationships that could be construed as a potential conflict of interest.

## Publisher’s note

All claims expressed in this article are solely those of the authors and do not necessarily represent those of their affiliated organizations, or those of the publisher, the editors and the reviewers. Any product that may be evaluated in this article, or claim that may be made by its manufacturer, is not guaranteed or endorsed by the publisher.
